# Cardiovascular adverse events associated with cyclophosphamide, pegylated liposomal doxorubicin, vincristine, and prednisone with or without rituximab ((R)-CDOP) in non-Hodgkin’s lymphoma: A systematic review and meta-analysis

**DOI:** 10.3389/fphar.2022.1060668

**Published:** 2022-12-01

**Authors:** Bin Lu, Longfei Shen, Ying Ma, Jia Qi, Yulin Li, Zhihao Wang, Lu Han, Ming Zhong

**Affiliations:** ^1^ The Key Laboratory of Cardiovascular Remodeling and Function Research, Chinese Ministry of Education, Chinese National Health Commission and Chinese Academy of Medical Sciences, The State and Shandong Province Joint Key Laboratory of Translational Cardiovascular Medicine, Department of Cardiology, Qilu Hospital, Cheeloo College of Medicine, Shandong University, Jinan, Shandong, China; ^2^ Shandong Key Laboratory of Cardiovascular Proteomics, Department of Geriatric Medicine, Qilu Hospital, Cheeloo College of Medicine, Shandong University, Jinan, Shandong, China; ^3^ Department of Cardiology, Zibo Central Hospital, Zibo, Shandong, China; ^4^ Department of General Practice, Qilu Hospital, Cheeloo College of Medicine, Shandong University, Jinan, Shandong, China

**Keywords:** non-Hodgkin’s lymphoma, liposomes, (R)-CDOP combination regimen, cardiotoxicity, adverse cardiovascular events

## Abstract

**Background:** The (R)-CDOP combination regimen, based on pegylated liposomal doxorubicin, is increasingly used for elderly patients with non-Hodgkin’s lymphoma. However, the cardiotoxicity and efficacy of the (R)-CDOP regimen compared with conventional anthracyclines have not been demonstrated in the general population. Therefore, this systematic review and meta-analysis evaluated the risk of cardiotoxicity and efficacy associated with the (R)-CDOP regimen in patients with non-Hodgkin’s lymphoma.

**Methods:** PubMed, Embase, Cochrane Library, CNKI, WanFang Database, and VIP were searched. The search covered the period from the start of the clinical use of (R)-CDOP to April 2022. We searched the literature for cardiovascular adverse events associated with (R)-CDOP in non-Hodgkin’s lymphoma. The data were analyzed using R 4.2.0 and Stata 12.0.

**Results:** From the included studies, the important findings were as follows: total cardiovascular event rate, 7.45% (95% confidence interval [CI] = 4.86%–10.44%); non-serious cardiovascular adverse event rate, 6.48% (95% CI = 3.70%–9.8%); serious cardiovascular adverse event rate, 0.67% (95% CI = 0.00%–2.12%); heart failure rate, 0.55% (95% CI = 0.00%–1.93%); rate of treatment discontinuation attributable to left ventricular dysfunction or heart failure, 0.02% (95% CI = 0.00%–0.57%); and cardiovascular death rate, 0.00% (95% CI = 0.00%–0.37%). Compared with the (R)-CHOP regimen, the (R)-CDOP regimen reduced the risk of cardiovascular events, including total cardiovascular adverse events (odds ratio [OR] = 0.161, 95% CI = 0.103–0.251, *p* < 0.001, and NNT = 3.7), non-serious cardiovascular adverse events (OR = 0.171, 95% CI = 0.093–0.314, *p* < 0.001, and NNT = 3.6), serious cardiovascular adverse events (OR = 0.252, 95% CI = 0.119–0.535, *p* < 0.001, and NNT = 6.8), and heart failure (OR = 0.294, 95% CI = 0.128–0.674, *p* = 0.004, and NNT = 9.5). To evaluate the survival benefits, we compared (R)-CDOP and (R)-CHOP regimens. We found that the (R)-CDOP regimen was no less efficacious, including complete remission (CR) (OR = 1.398, 95% CI = 0.997–1.960, and *p* = 0.052), partial response (PR) (OR = 1.631, 95% CI = 1.162–2.289, and *p* = 0.005), objective response rate (ORR) (OR = 2.236, 95% CI = 1.594–3.135, and *p* < 0.001), stable disease (SD) (OR = 0.526, 95% CI = 0.356–0.776, and *p* = 0.001), and progressive disease (PD) (OR = 0.537, 95% CI = 0.323–0.894, and *p* = 0.017).

**Conclusion:** Our findings suggested that the (R)-CDOP regimen had a lower risk of cardiovascular adverse events in non-Hodgkin’s lymphoma than the (R)-CHOP regimen, demonstrating its safety with regard to cardiotoxicity. In addition, this study found the (R)-CDOP regimen was no less efficacious than the (R)-CHOP regimen in the treatment of non-Hodgkin’s lymphoma. These findings need to be validated by higher-quality research because of the limited number and quality of included studies.

## Introduction

Non-Hodgkin’s lymphoma (NHL) is a lymphoid malignancy with a variety of biological and clinical behaviors, usually involving lymphatic and hematopoietic tissues but also other organs (Ganapathi, Brown, Prakash, & Bhargava, 2021). Most patients usually have persistent painless lymphadenopathy, but some patients also develop systemic symptoms such as night sweats, persistent fever, and unexplained weight loss (Bowzyk Al-Naeeb, Ajithkumar, Behan, & Hodson, 2018). NHL is the most common hematological malignancy worldwide, accounting for nearly 3% of all cancer diagnoses, and its average age of diagnosis is 67 years (Thandra et al., 2021). Patients with NHL are generally elderly, and they often have cardiovascular risk factors or a history of heart disease, making them more susceptible to cardiotoxicity caused by chemotherapeutic drugs (Abuamsha, Kadri, & Hernandez, 2019). In addition to being a reason for treatment discontinuation, severe cardiotoxicity may be a reason for serious or even life-threatening events.

The (R)-CHOP (rituximab, cyclophosphamide, vincristine, doxorubicin, and prednisone) regimen, an anthracycline-based regimen, is often the first-line treatment for NHL, particularly for diffuse large B-cell lymphoma (Sehn & Salles, 2021). Cardiotoxicity is a particular complication of anthracyclines. The cumulative dose of anthracyclines has been reported consistently as an important factor causing cardiotoxicity. Swain SM et al. found that if the dosage of anthracyclines was greater than 550 mg/m^2^, it would cause relevant cardiomyopathy in 26% of patients (Swain, Whaley, & Ewer, 2003). At present, the main measure to prevent related cardiotoxicity is to reduce the cumulative dose to less than 450 mg/m^2^, but this also limits efficacy and cannot prevent all cardiovascular adverse events (Jones et al., 2021). Due to the uncertainty of the predictors of cardiotoxicity, the optimal monitoring strategy has not been agreed upon. Therefore, the individual management of chemotherapy drugs is still challenging. Doxorubicin liposome is a nanoparticle-based anti-tumor drug approved by the FDA and has been widely used to treat a variety of tumors (Herrmann et al., 2022). However, their clinical application has been limited by their structural instability, drug leakage, short shelf life, and poor tissue targeting. Pegylated liposomal doxorubicin (PLD) is a new type of liposome, in which macromolecular polyethylene glycol (PEG) is coated with doxorubicin on the surface, which makes the release rate of doxorubicin from the heart much lower than that from other tissues, effectively reducing the cardiotoxicity of doxorubicin (O'Brien et al., 2004; Theodoulou & Hudis, 2004). PLD is increasingly used to treat elderly patients with NHL, providing a new strategy for elderly patients. Despite the good safety of PLD and the widespread use of (R)-CDOP in the population, it is unknown whether the use of multiple drugs increases the risk of cardiotoxicity compared with that of PLD alone.

To obtain better scientific evidence, we conducted a meta-analysis to assess the risk of cardiovascular adverse events associated with the (R)-CDOP regimen. Furthermore, we explored the cardiovascular adverse event risk and efficacy of the (R)-CDOP regimen compared with the (R)-CHOP regimen.

## Methods

### Search strategy and study selection

PubMed, Embase, Cochrane Library, CNKI, WanFang Database, and VIP were searched. The search covered the period from the start of the clinical use of the (R)-CDOP regimen to April 2022. English databases were searched using combinations of the following keywords: “lymphoma,” “pegylated liposomal doxorubicin,” and “liposome.” The search terms for Chinese databases included “lymphoma” and “liposome.” The complete search string is provided in the Supplementary Appendix S1 (**p 1**). There was no language restriction. Literature tracing was performed using the references in the included studies. The complete inclusion and exclusion criteria are provided in the Supplementary Appendix S1 (**p 2**).

### Outcomes

The outcomes included non-serious and serious cardiovascular adverse events. Non-serious cardiovascular adverse events included grade 1–2 cardiovascular adverse events (preferably using a well-established toxicity grading system to quantify severity) and grade 1–2 cardiovascular adverse events according to the CTCAE 5.0 (if the cardiovascular adverse events were not graded). Cardiovascular adverse events were considered serious if at least one of the following outcomes could be extracted: grade 3–4 cardiovascular adverse events (preferably using a well-established toxicity grading system to quantify severity), grade 3–4 cardiovascular adverse events according to the CTCAE 5.0 (if the cardiovascular adverse events were not graded), heart failure (10% decrease in the LVEF from the baseline to <53% (Herrmann, 2020)), cardiac function (grade III + IV), interruption of therapy because of the left ventricular dysfunction or heart failure, and treatment-related cardiovascular death.

### Data extraction, evaluation, and synthesis

Two reviewers independently completed the literature search and literature screening using EndNote X9 literature management software. The reviewers used a pre-designed Excel sheet for literature extraction. Additional data were collected from the eligible studies, including first author, publication time, study region, study type, median age, the proportion of female patients, disease subtype, treatment period, treatment interval, median follow-up time, cardiotoxicity grading system, and outcome indicators. Disagreements throughout the process were resolved with a third reviewer.

### Quality assessment in individual studies

The methodological index for non-randomized controlled studies (MINORS) was used to evaluate the quality of the included studies (Slim et al., 2003). For this index, studies with scores of 0–8 points were graded C, those with scores of 9–16 points were graded B, and those with scores of 17–24 points were graded A. The classification indices are described in detail in the Supplementary Appendix S1. The complete evaluation is detailed in the Supplementary Appendix S1 (**p 3**).

### Risk of bias

Since the included studies primarily assessed the effectiveness of chemotherapy regimens rather than the occurrence of adverse effects, incomplete outcome data and selective reporting were considered important potential sources of bias. Therefore, we did not use the traditional Cochrane risk-of-bias tool to assess bias in the studies. Since the outcomes of this study were cardiovascular adverse events, we performed a quality assessment for cardiovascular adverse events (Linschoten et al., 2020). Specific information on the risk of bias is presented in the Supplementary Appendix S1 (p 4). The funnel plots of the publication bias have been included, which allows a better evaluation of the homogeneity of its distribution. Specific information on the risk of publication bias is presented in the appendix (p 5).

### Statistical analyses

All statistical analyses were conducted using R (version 4.2.0) and Stata (version 12.0). To estimate the incidence of cardiovascular adverse events in patients treated with (R)-CDOP, a double-arcsine transformation was used to obtain a normal distribution appropriate for pooling, with the proportion estimates expressed as 95% confidence intervals (CIs). The (R)-CDOP regimen was compared with the (R)-CHOP regimen. Dichotomous variables were expressed as odds ratios (ORs) and 95% CIs. The study heterogeneity was assessed using the Q-test, with I^2^ > 50% indicating high heterogeneity and I^2^ < 50% indicating low heterogeneity.

## Results

**FIGURE 1:**
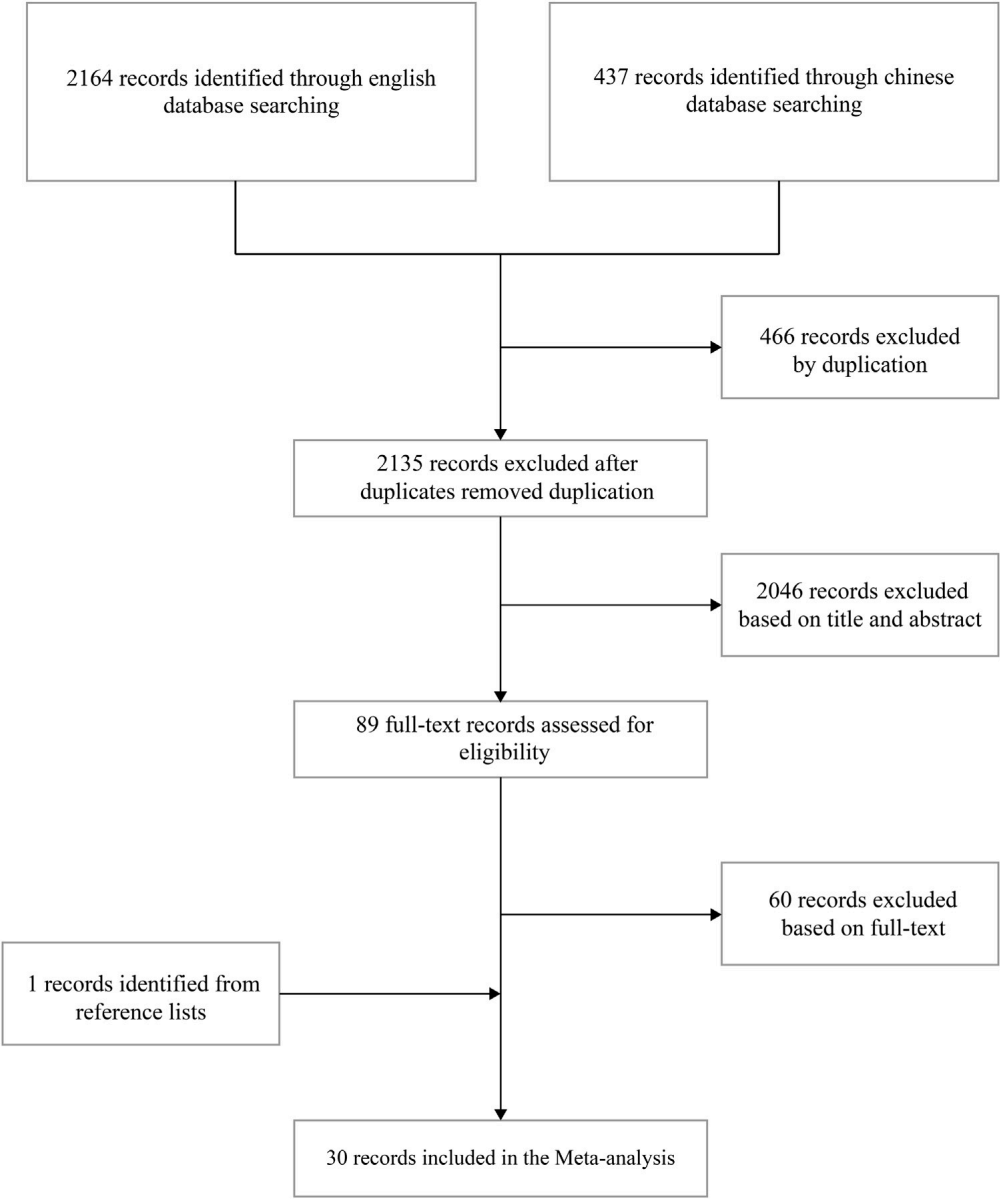
Study flow diagrams.

In total, 2,601 studies were retrieved (PubMed, *n* = 30; Embase, *n* = 599; Cochrane Library, *n* = 1,535; CNKI, *n* = 181; WanFang Database, *n* = 181; and VIP, *n* = 75). Through literature screening, 30 studies were evaluated for eligibility. The specific literature screening process is presented in Figure 1.

### General characteristics of studies included in the meta-analysis

We conducted a meta-analysis to examine the incidence of cardiovascular adverse events associated with the (R)-CDOP regimen. Thirty publications involving 1,071 patients were included. The basic characteristics of the study population are presented in Table 1. To further compare cardiovascular adverse event rates between the (R)-CDOP and (R)-CHOP regimens, 11 publications with a total of 381 patients in the (R)-CDOP group and 374 patients in the (R)-CHOP group were included. The main population characteristics are presented in Table 2.

**TABLE 1:** Baseline characteristics of single-arm studies included.

Reference	Country	Study type	N	Median age (years)	Female (%)	Population	Cycles (n)	Interval (days)	Median follow-up (months)	Toxicity grading system	Outcome indicators
Liu and Zhang (2021)	China	Retrospective cohort	40	67	48	DLBCL	6	21	36	Grades NOS	**①②③④**
Zaja et al. (2006)	Italy	Single-arm trial	29	69	45	DLBCL	6	21	24	WHO	**①②③④⑤⑥**
Martino, R (2002)	Spain	Single-arm trial	33	74	61	DLBCL	6	21	13	WHO	**①②③④⑤⑥**
Aviles, A (2002)	Mexico	Single-arm trial	20	65	60	DLBCL	6	21	18.1	WHO	**①②③④⑤⑥**
Tsavaris, N (2002)	Greece	Single-arm trial	25	79	36	NHL	6	21	12	WHO	**①②③④⑤⑥**
Oki et al. (2015)	America	Single-arm trial	80	69	NR	DLBCL	6–8	21	46	CTCAE 2.0	**①③④⑤⑥**
Schmitt, C. J (2012)	Germany	Retrospective cohort	21	68	NR	NHL	4	NR	20	CTCAE	**①②③④⑤⑥**
Zhou et al. (2015)	China	Retrospective cohort	41	67	51	DLBCL	6	21	28	CTCAE 3.0	**①③④**
Visani, G (2005)	Italy	Cohort	13	69	38	NHL	6	21	18	WHO	**①②③④⑤⑥**
Fan et al. (2011)	China	Cohort	14	36	36	PTCL	6	21	15	CTCAE 3.0	**①②③④⑤⑥**
Yang, F.L (2019)	China	Retrospective cohort	94	61	54	DLBCL	6	21	10	Grades NOS	**①②③④⑤⑥**
Shen, W.N (2016)	China	Cohort	21	49	33	NHL	6	NR	NR	CTCAE 4.0	**①②③④⑤⑥**
Lin et al. (2020)	China	Retrospective cohort	94	70	43	DLBCL	4.5	21	61	CTCAE 4.0	**①②③⑤⑥**
Shao et al. (2020)	China	Retrospective cohort	33	63	46	DLBCL	8	21	24	Grades NOS	**①②③④**
Zheng et al. (2018)	China	Cohort	21	62	48	NHL	6	21	36	Grades NOS	**①②③④**
Hu et al. (2018)	China	Cohort	30	71	45	NHL	3–4	21	24	Grades NOS	**①**
Jia et al. (2017)	China	Retrospective cohort	26	70	31	DLBCL	NR	21	NR	CTCAE 3.0	**①②③④⑤⑥**
Shen et al. (2020)	China	Retrospective cohort	23	NR	83	NHL	NR	21	NR	Grades NOS	**①**
Li et al. (2016)	China	Cohort	25	69	44	DLBCL	4–6	21	18	Grades NOS	**①②③④⑤⑥**
Huang and Lou (2016)	China	Cohort	25	47	50	DLBCL	6–8	21	24	CTCAE 4.0	**①⑤**
Huang et al. (2021)	China	Cohort	15	55	47	NHL	4	NR	NR	Grades NOS	**①**
Guo et al. (2009)	China	Cohort	34	84	6	NHL	5.2	21	NR	WHO	**①②③④⑤⑥**
Wang et al. (2021)	China	Retrospective cohort	31	83	7	DLBCL	7	21–28	36	CTCAE 4.0	**①②③④⑤⑥**
Ye (2022)	China	Cohort	44	48	46	NHL	NR	NR	6	Grades NOS	**①**
Chang and Zhu (2021)	China	Retrospective cohort	37	57	54	DLBCL	NR	21	NR	CTCAE 5.0	**①②③④**
Gui et al. (2015)	China	Cohort	30	70	63	DLBCL	6	21	20.1	CTCAE 4.0	**①②③④⑤⑥**
Li and Hu (2018)	China	Cohort	34	NR	NR	DLBCL	6	21	NR	WHO	**①②③④⑤⑥**
Wu et al. (2020)	China	Retrospective cohort	23	56	52	DLBCL	2	21	NR	CTCAE 5.0	**①②③④⑤⑥**
Song and Ding (2014)	China	Cohort	32	66	NR	DLBCL	6	21	NR	Grades NOS	**①②③④⑤⑥**

Abbreviations: DLBCL, diffuse large B-cell lymphoma; NHL, non-Hodgkin’s lymphoma; PTCL, peripheral T-cell lymphoma; NR, not reported; CTCAE, Common Terminology Criteria for Adverse Events; WHO, World Health Organization; NOS, not otherwise specified; and outcome indicators (① total cardiovascular adverse events; ② non-serious cardiovascular adverse events; ③ serious cardiovascular adverse events; ④ heart failure; ⑤ treatment discontinuations due to an adverse cardiovascular event; and ⑥ treatment-related cardiovascular deaths).

**TABLE 2:** Baseline characteristics of studies with the control group.

Reference	Area	(R)-CDOP			(R)-CHOP			Population	Cycles (n)	Interval (days)	Median follow-up (months)	Toxicity grading system	Outcome indicators
		N	Median age (years)	Female (%)	N	Median age (years)	Female (%)						
Zhou (2015)	China	41	67	51	62	68	45	DLBCL	6	21	28	CTCAE 3.0	①③④
Shao et al. (2020)	China	33	63	46	45	61	47	DLBCL	8	21	24	Grades NOS	①②③④
Zheng et al. (2018)	China	21	62	48	21	62	43	NHL	6	21	36	Grades NOS	①②
Li et al. (2016)	China	25	69	44	25	68	40	DLBCL	4–6	21	18	Grades NOS	①②③④
Huang and Luo (2016)	China	25	47	50	25	44	60	DLBCL	6–8	21	24	CTCAE 4.0	①
Huang et al. (2021)	China	15	54	47	15	54	40	NHL	4	NR	NR	Grades NOS	①
Ye, J (2022)	China	44	48	46	44	46	50	NHL	NR	NR	6	Grades NOS	①
Chang and Zhu (2021)	China	37	57	54	39	51	67	DLBCL	NR	21	NR	CTCAE 5.0	①②
Li and Hu (2018)	China	34	NR	NR	30	NR	NR	DLBCL	6	21	NR	WHO	①②③④
Wu et al. (2020)	China	23	56	52	23	52	61	DLBCL	2	21	NR	CTCAE 5.0	①②③④
Liu et al. (2021)	China	83	69	59	45	70	44	PTCL	6	21	12	Grades NOS	①

Abbreviations: DLBCL, diffuse large B-cell lymphoma; NHL, non-Hodgkin’s lymphoma; PTCL, peripheral T-cell lymphoma; NR, not reported; CTCAE, Common Terminology Criteria for Adverse Events; WHO, World Health Organization; NOS, not otherwise specified; and outcome indicators (① total cardiovascular adverse events; ② non-serious cardiovascular adverse events; ③ serious cardiovascular adverse events; and ④ heart failure).

### Literature evaluation

We assessed the quality of 30 studies using the MINORS scale. Sixteen studies were single‐arm studies without a control group, and 14 studies had a control group. In total, 9, 19, and 2 studies were graded A, B, and C, respectively. The results of the quality assessment are presented in the Supplementary Appendix S1 (p 6). The results of the risk of bias are also presented in the Supplementary Appendix S1 (p 7 and p 8).

## Meta-analysis for cardiovascular adverse events

### Incidence of cardiovascular adverse events with the (R)-CDOP combination regimen

**FIGURE 2:**
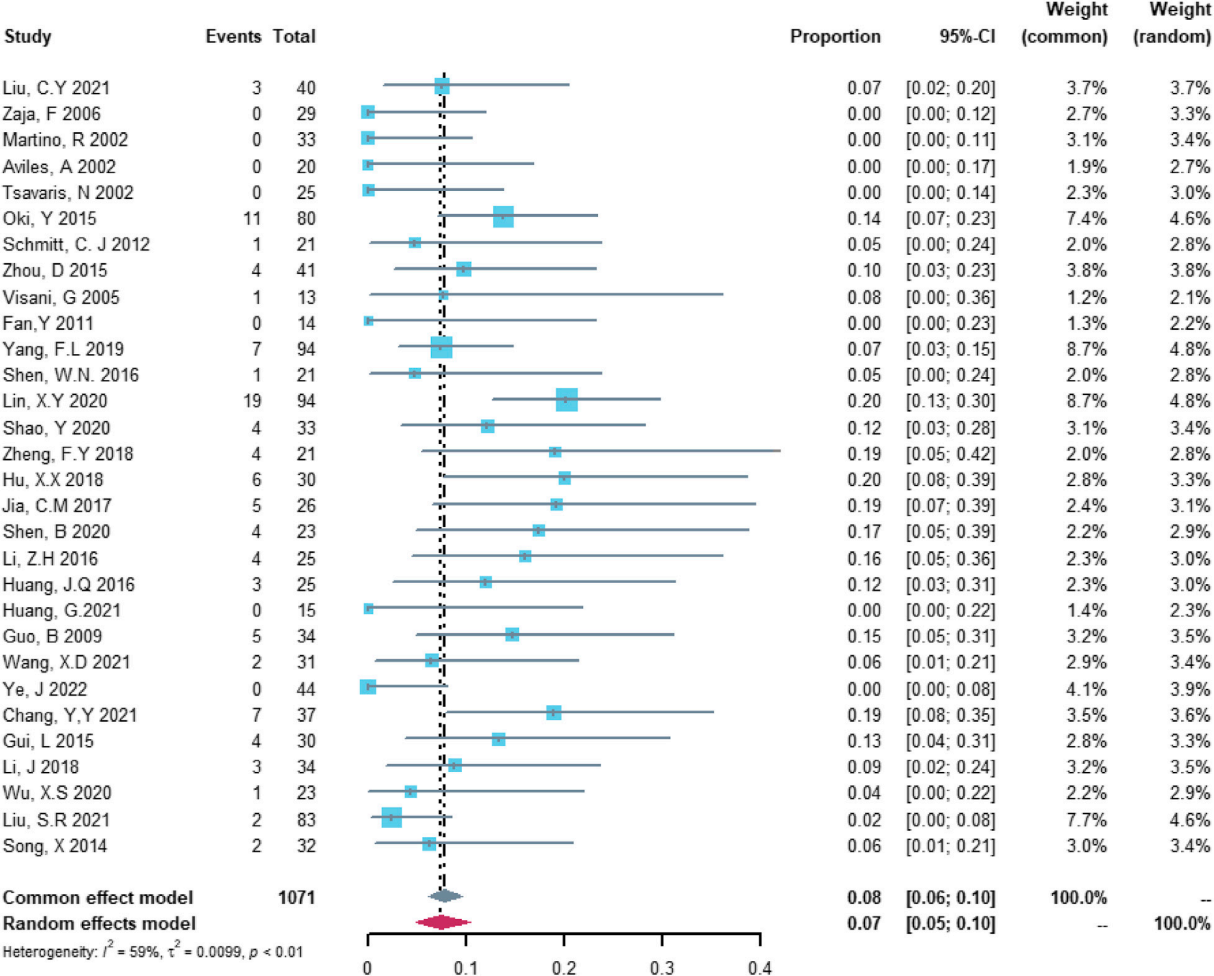
Summary pooled proportion analysis of total cardiovascular adverse events associated with the (R)-CDOP combination regimen.

In the analysis of total cardiovascular adverse events, 30 studies with 1,071 patients were included in the analysis. The study heterogeneity was high (τ^2^ = 0.0099, I^2^ = 59.2%, and *p* < 0.0001). The total cardiovascular adverse event rate was 7.45% (95% CI = 4.86%–10.44%; Figure 2).

**FIGURE 3:**
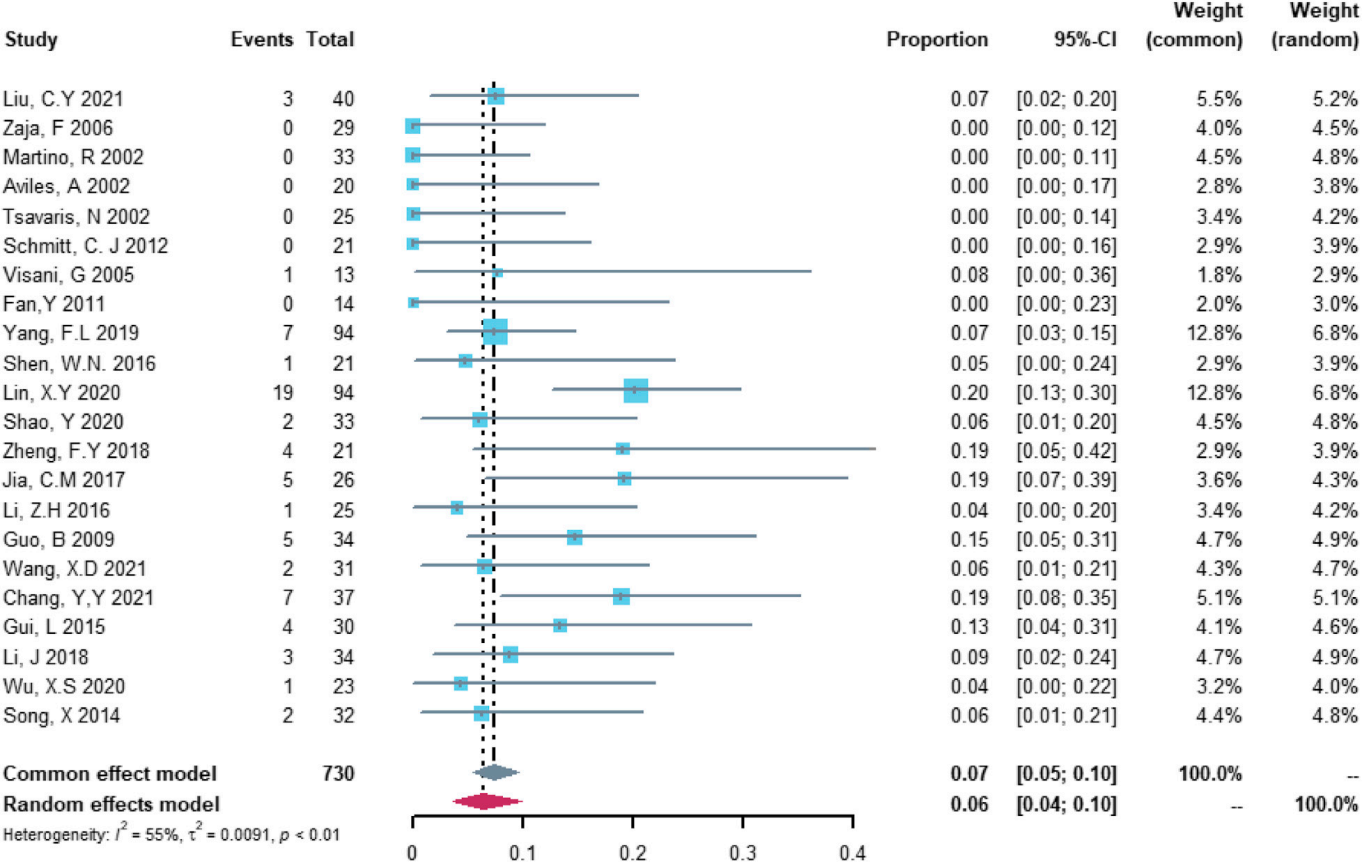
Summary pooled proportion analysis of non-serious cardiovascular adverse events associated with the (R)-CDOP combination regimen.

In the analysis of non-serious cardiovascular adverse events, 22 studies with 730 patients were included. The study heterogeneity was high (τ^2^ = 0.0091, I^2^ = 55.5%, and *p* = 0.0009). The non-serious cardiovascular adverse event rate was 6.48% (95% CI = 3.70%–9.8%; Figure 3).

**FIGURE 4:**
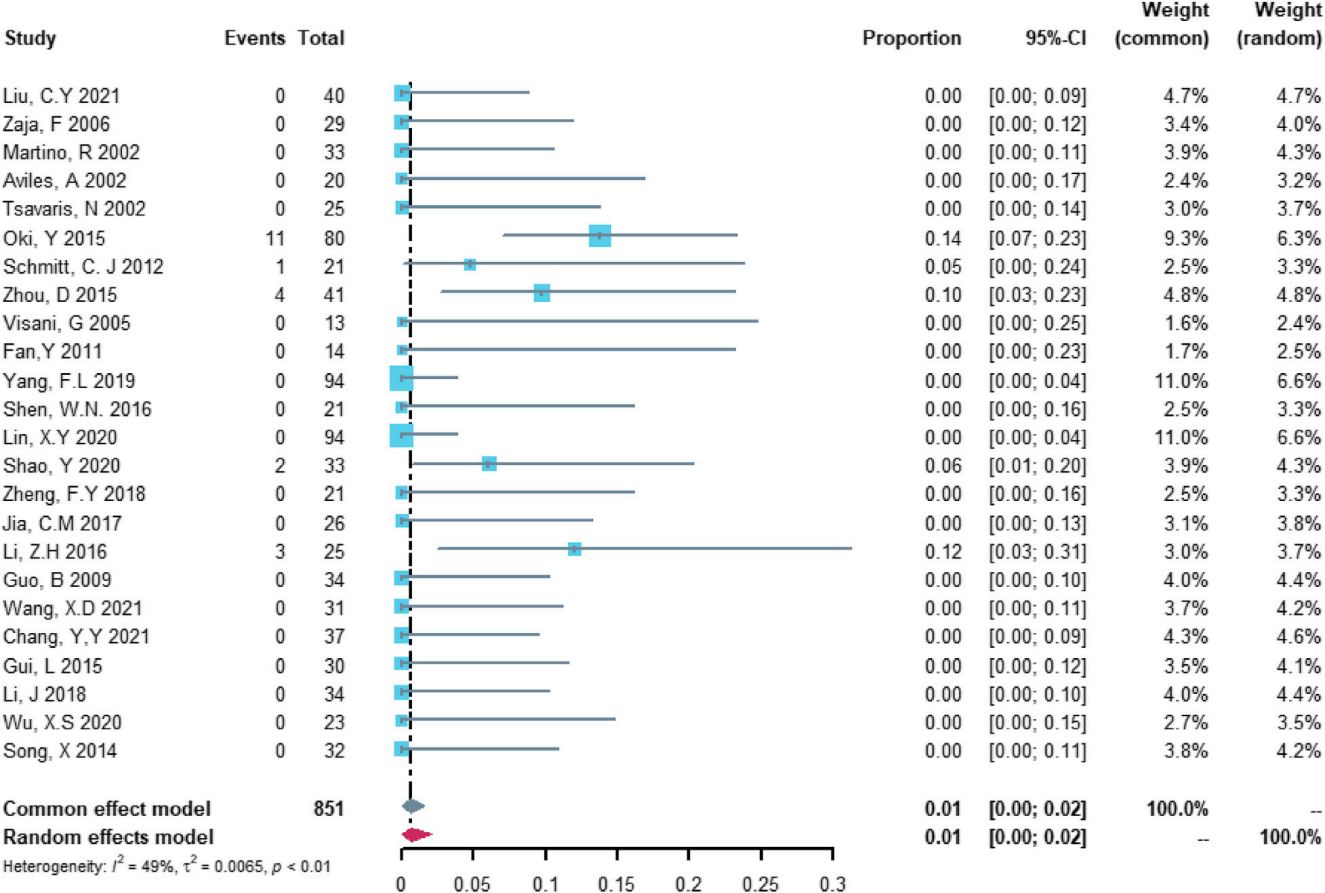
Summary pooled proportion analysis of serious cardiovascular adverse events associated with the (R)-CDOP combination regimen.

Twenty-four studies with 851 patients were included in the analysis of serious cardiovascular adverse events. The study heterogeneity was low (τ^2^ = 0.0065, I^2^ = 49.2%, and *p* = 0.0037). The serious cardiovascular adverse event rate was 0.67% (95% CI = 0.00%–2.12%; Figure 4).

**FIGURE 5:**
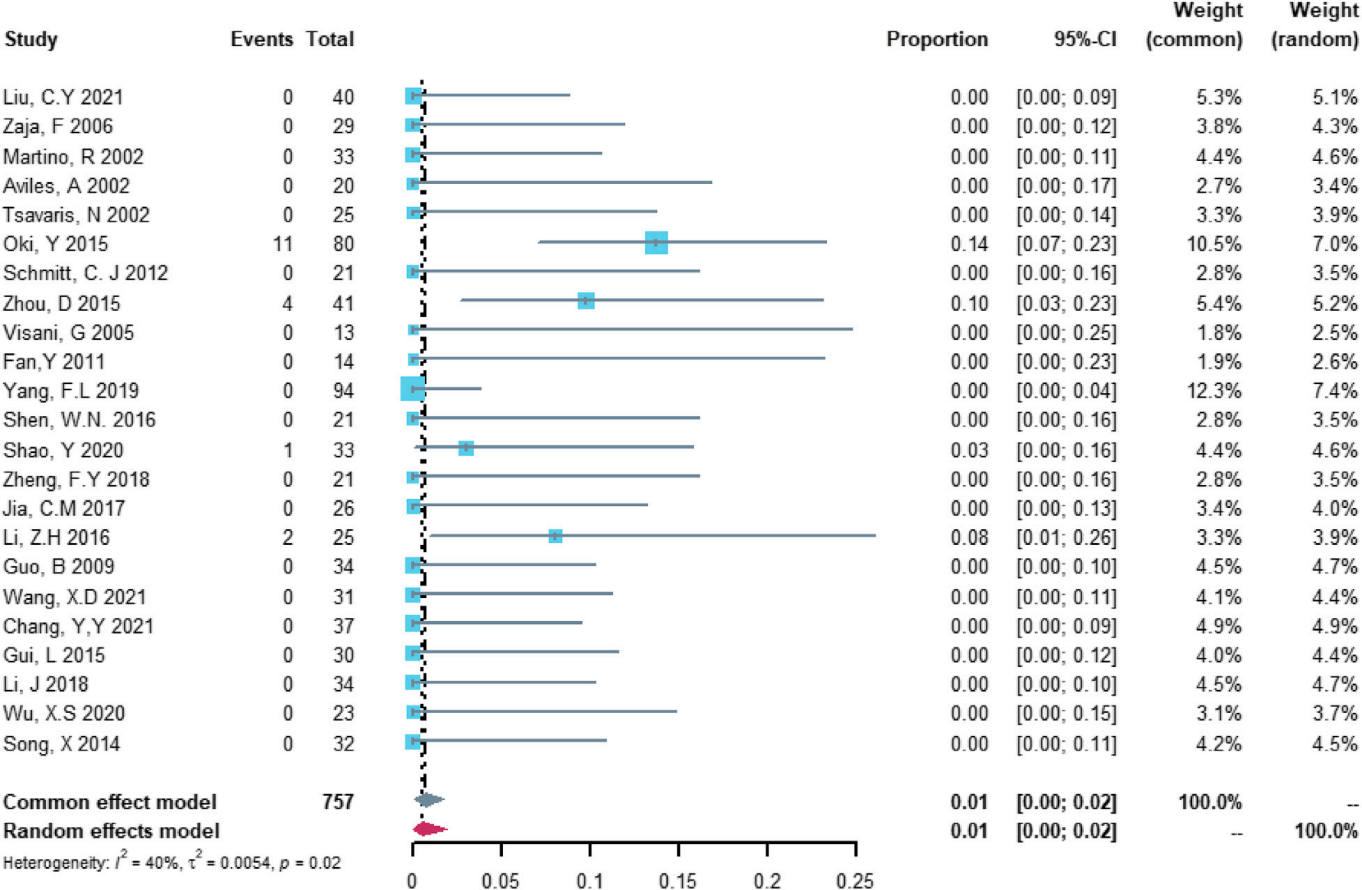
Summary pooled proportion analysis of heart failure cardiovascular adverse events associated with the (R)-CDOP combination regimen.

Twenty-three studies with 757 patients were included in the analysis of heart failure. The study heterogeneity was low (τ^2^ = 0.0054, I^2^ = 40.2%, and *p* = 0.0249). The heart failure rate was 0.55% (95% CI = 0.00%–1.93%; Figure 5).

**FIGURE 6:**
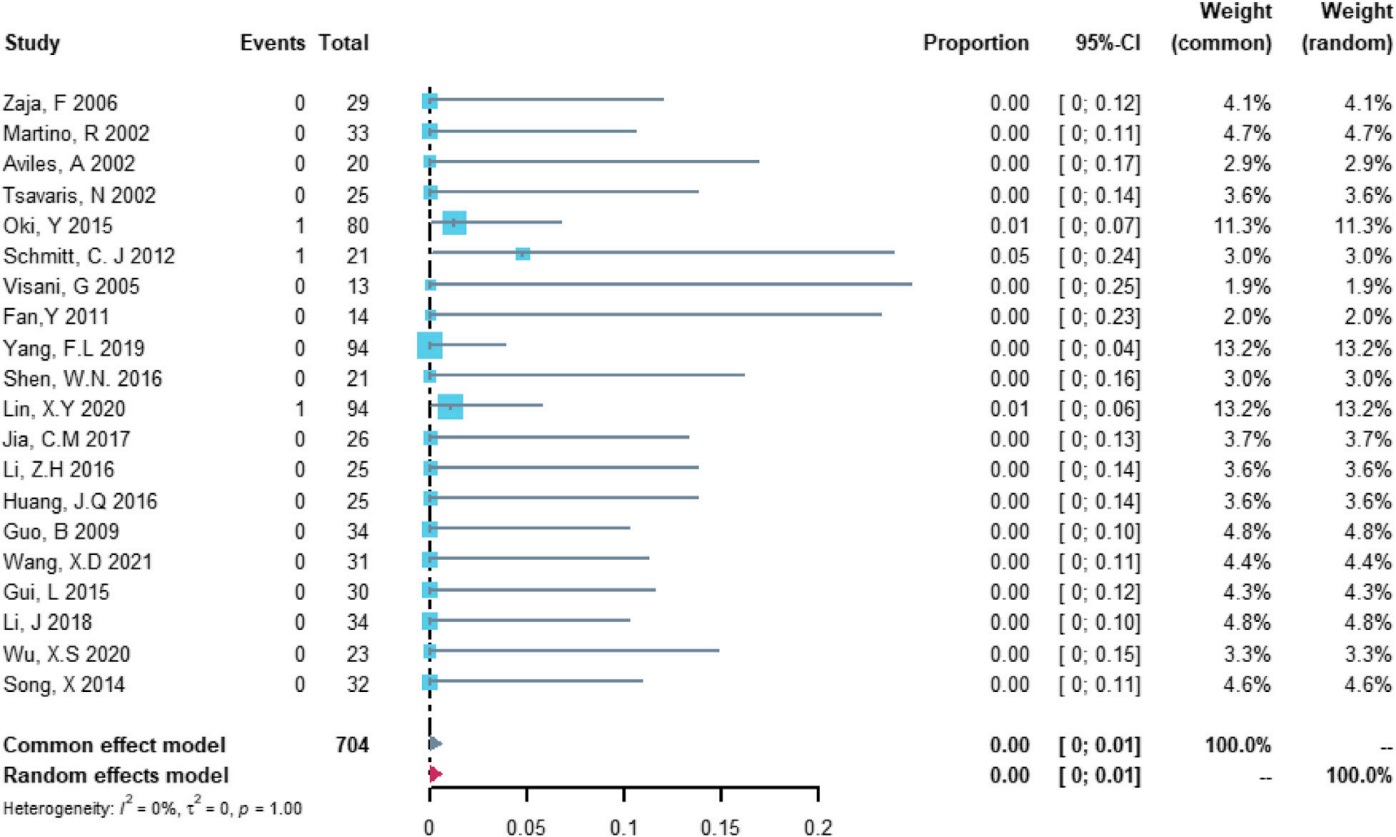
Summary pooled proportion analysis of treatment discontinuation events due to the left ventricular dysfunction or heart failure associated with the (R)-CDOP combination regimen.

Twenty studies with 704 patients were included in the analysis of treatment discontinuation attributable to left ventricular dysfunction or heart failure. The study heterogeneity was low (τ^2^ = 0.0000, I^2^ = 0.0%, and *p* = 0.9999). The treatment discontinuation rate was 0.02% (95% CI = 0.00%–0.57%; Figure 6).

**FIGURE 7:**
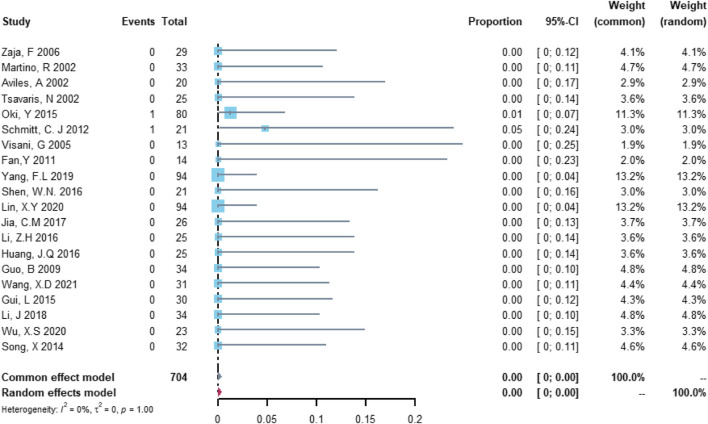
Summary pooled proportion analysis of cardiovascular death events associated with the (R)-CDOP combination regimen.

Twenty studies with 704 patients were included in the analysis of cardiovascular death. The study heterogeneity was low (τ^2^ = 0.0000, I^2^ = 0.0%, and *p* = 0.9997). The cardiovascular death rate was 0.00% (95% CI = 0.00%–0.37%; Figure 7).

### Comparison of the risk of cardiovascular adverse events between the (R)-CDOP and (R)-CHOP regimens

**FIGURE 8:**
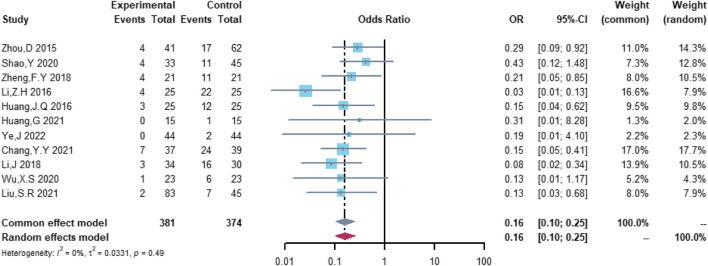
Changes in total cardiovascular adverse events in (R)-CDOP *vs.* (R)-CHOP treatment.

Eleven studies were included in the analysis of total cardiovascular adverse events, including 381 patients in the (R)-CDOP group and 374 patients in the (R)-CHOP group. The study heterogeneity was low (τ^2^ = 0.0331, I^2^ = 0.0%, and *p* = 0.487). (R)-CDOP was linked to a lower risk of total cardiovascular adverse events than (R)-CHOP (OR = 0.161, 95% CI = 0.103–0.251, *p* = 0.000, and NNT = 3.7; Figure 8).

**FIGURE 9:**
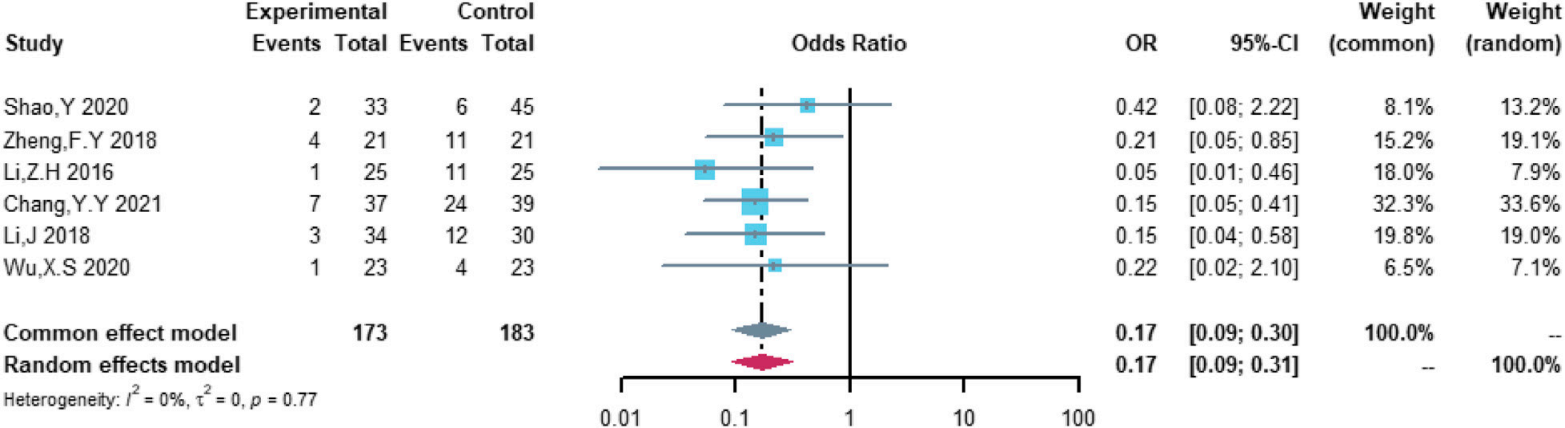
Changes in non-serious cardiovascular adverse events in (R)-CDOP *vs.* (R)-CHOP treatment.

A total of six studies were included in the analysis of non-serious cardiovascular adverse events, including 173 patients in the (R)-CDOP group and 183 patients in the (R)-CHOP group. The study heterogeneity was low (τ^2^ = 0.0000, I^2^ = 0.0%, and *p* = 0.770). (R)-CDOP reduced the risk of non-serious cardiovascular adverse events *vs.* (R)-CHOP (OR = 0.171, 95% CI = 0.093–0.314, *p* = 0.000, and NNT = 3.6; Figure 9).

**FIGURE 10:**
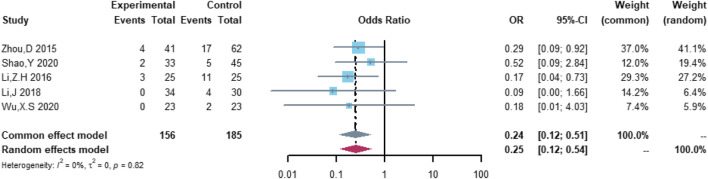
Changes in serious cardiovascular adverse events in (R)-CDOP *vs.* (R)-CHOP treatment.

Five studies were included in the analysis of serious cardiovascular adverse events, including 156 patients in the (R)-CDOP group and 185 patients in the (R)-CHOP group. The study heterogeneity was low (τ^2^ = 0.0000, I^2^ = 0.0%, and *p* = 0.819). (R)-CDOP reduced the risk of serious cardiovascular adverse events *vs.* (R)-CHOP (OR = 0.252, 95% CI = 0.119–0.535, *p* = 0.000, and NNT = 6.8; Figure 10).

**FIGURE 11:**
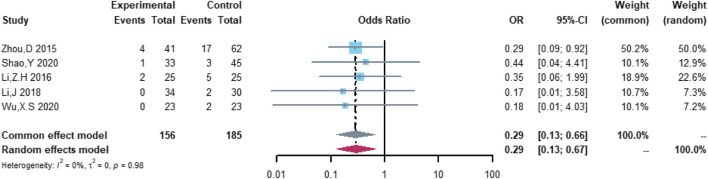
Changes in heart failure adverse events in (R)-CDOP *vs.* (R)-CHOP treatment.

Five studies were included in the analysis of heart failure, including 156 patients in the (R)-CDOP group and 185 patients in the (R)-CHOP group. The study heterogeneity was low (τ^2^ = 0.0000, I^2^ = 0.0%, and *p* = 0.984). (R)-CDOP was linked to a lower risk of heart failure than (R)-CHOP (OR = 0.294, 95% CI = 0.128–0.674, *p* = 0.004, and NNT = 9.5; Figure 11).

### Comparison of the efficacy between the (R)-CDOP and (R)-CHOP regimens

**FIGURE 12:**
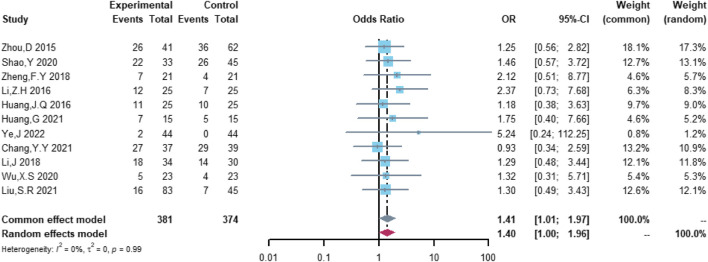
Analysis of complete remission (CR) in (R)-CDOP *vs.* (R)-CHOP treatment.

**FIGURE 13:**
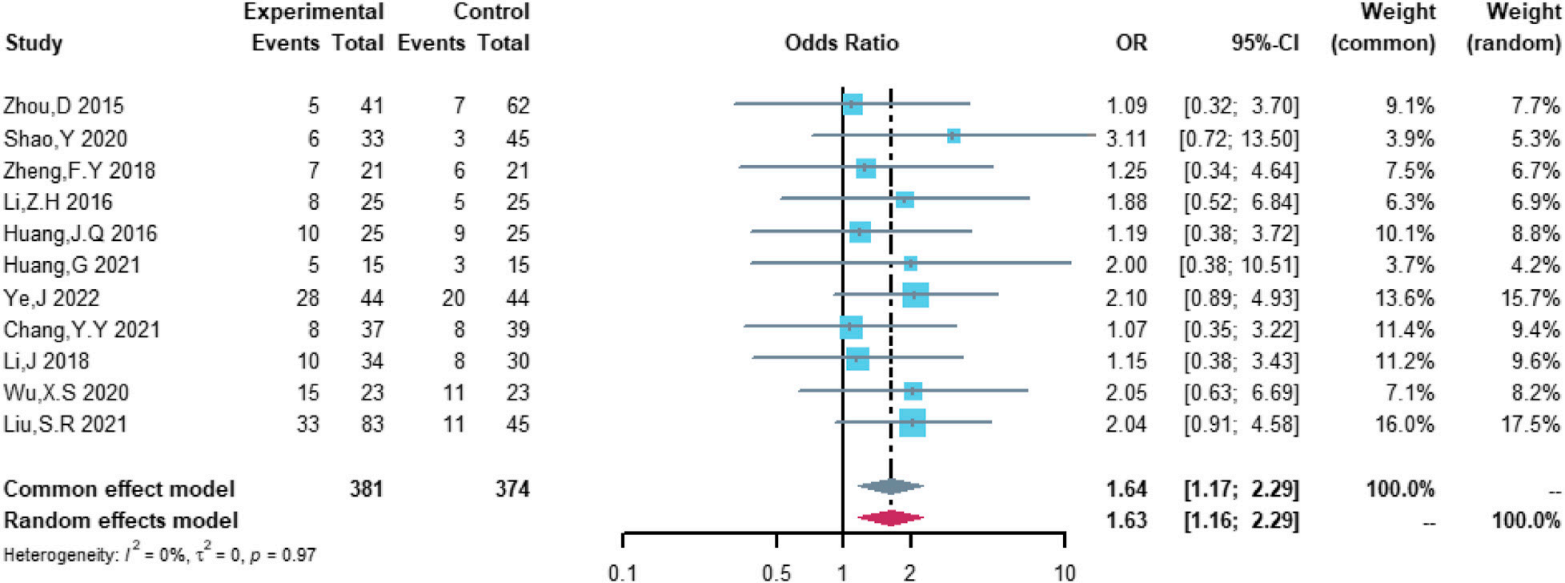
Analysis of partial response (PR) in (R)-CDOP *vs.* (R)-CHOP treatment.

**FIGURE 14:**
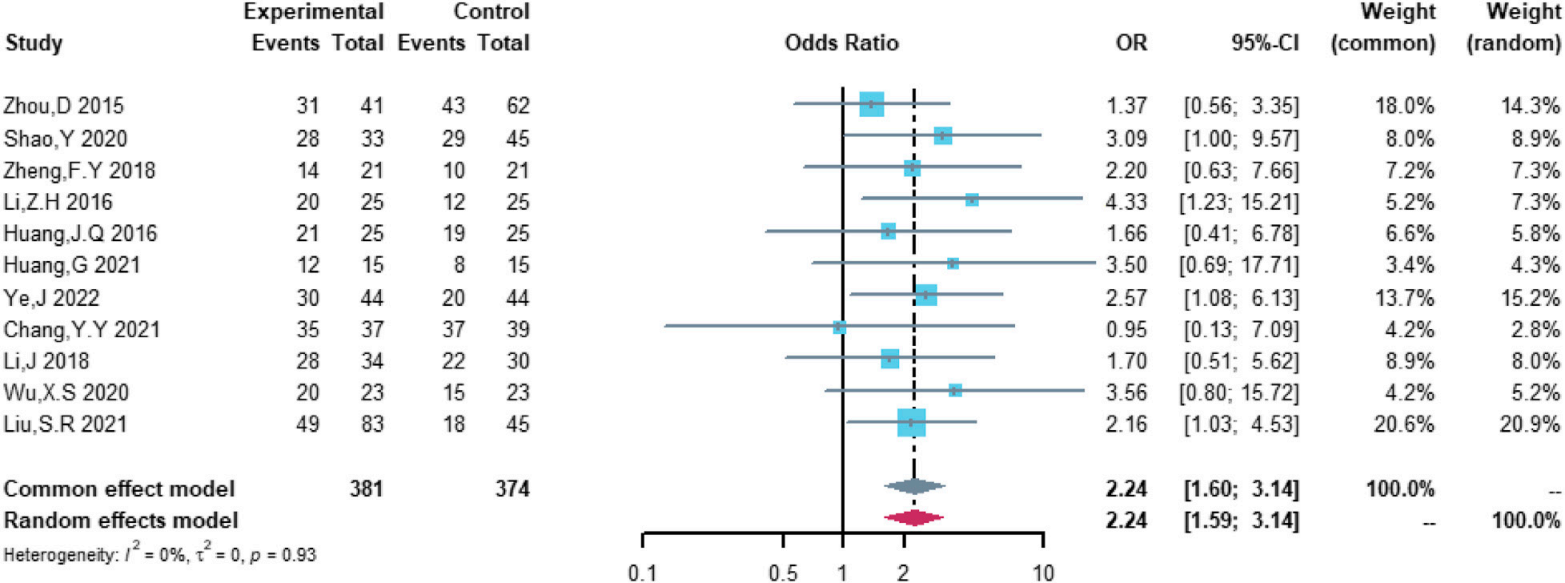
Analysis of objective response rate (ORR) in (R)-CDOP *vs.* (R)-CHOP treatment.

**FIGURE 15:**
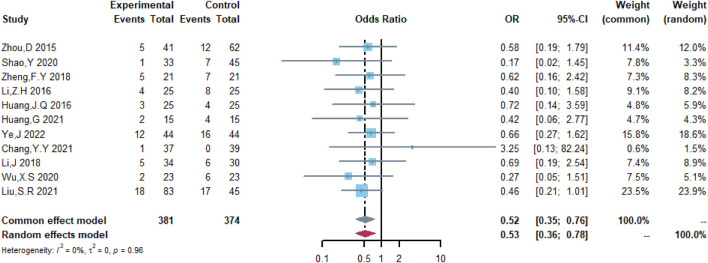
Analysis of stable disease (SD) in (R)-CDOP *vs.* (R)-CHOP treatment.

**FIGURE 16:**
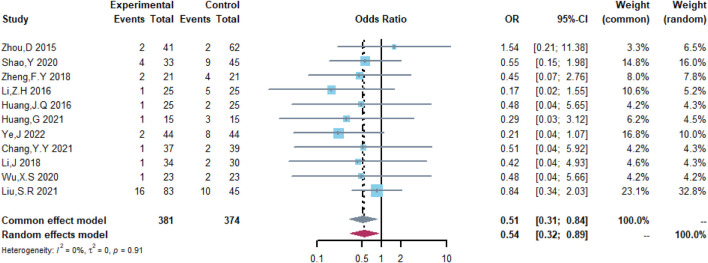
Analysis of progressive disease (PD) in (R)-CDOP *vs.* (R)-CHOP treatment.

Therefore, the study was designed to compare (R)-CDOP and (R)-CHOP regimens on clinical outcomes by systematically calculating the survival benefits. Eleven studies were included in the analysis of efficacy, including 381 patients in the (R)-CDOP group and 374 patients in the (R)-CHOP group. We found that the (R)-CDOP regimen is better than the (R)-CHOP regimen, but there was no significant difference in terms of CR (OR = 1.398, 95% CI = 0.997–1.960, and *p* = 0.052; Figure 12). The (R)-CDOP regimen had a better OR than the (R)-CHOP regimen (OR = 1.631, 95% CI = 1.162–2.289, and *p* = 0.005; Figure 13). We also found that the ORR had similar changes (OR = 2.236, 95% CI = 1.594–3.135, and *p* < 0.001; Figure 14). In addition, we evaluated the SD and PD, and then, we found that the (R)-CDOP regimen had a lower risk than the (R)-CHOP regimen in terms of SD and PD (OR = 0.526, 95% CI = 0.356–0.776, and *p* = 0.001, Figure 15; OR = 0.537, 95% CI = 0.323–0.894, and *p* = 0.017, Figure 16, respectively). These findings indicated that the (R)-CDOP regimen might maintain efficacy and could be considered for patients with NHL.

## Discussion

This study investigated the rate of cardiovascular adverse events with the (R)-CDOP regimen in patients with NHL. The study revealed that the (R)-CDOP combination carried a lower risk of cardiovascular adverse events than the (R)-CHOP regimen, indicating the cardiovascular safety of (R)-CDOP. In addition, the (R)-CDOP regimen might improve the prognosis compared with the (R)-CHOP regimen, providing more options for patients in the future.

Anthracyclines apply to the treatment of various types of cancers alone or in combination with other anti-tumor drugs. Mitochondrial dysfunction (Varricchi et al., 2018), disruption of calcium homeostasis (Tscheschner et al., 2019), and apoptosis-related protein production (Merten, Jiang, Feng, & Kang, 2006; Saleme et al., 2019) are important mechanisms of the cardiotoxic effects of anthracyclines. The cardiotoxicity caused by cumulative doses of these drugs is a major limiting factor in their application (Martins-Teixeira & Carvalho, 2020). A study showed that the incidence of left ventricular dysfunction was 10%, 16%, 32%, and 65% at the cumulative doses of doxorubicin of 250, 300, 400, and 550 mg/m^2^, respectively (Bernstein, 2018). According to the guidelines of the American Society of Clinical Oncology (ASCO), the cumulative dose of doxorubicin ≥ 250 mg/m^2^ is considered a high dose (Armenian et al., 2017). To mitigate this cardiotoxicity, novel anthracyclines are increasingly used in the treatment of cancer. Although the long-term cardiotoxicity of these drugs is unclear, studies revealed that anthracycline liposomes, particularly PLD, demonstrated cardiac safety for patients (X. R. Li, Cheng, Zhang, Wang, & Huang, 2022). Yamaguchi et al. revealed that doxorubicin liposomes were less cardiotoxic than conventional doxorubicin in a meta-analysis (Yamaguchi et al., 2015). L Ansari et al. also showed lower cardiotoxicity than traditional doxorubicin in women with metastatic breast cancer treated with pegylated liposomal doxorubicin alone (Ansari et al., 2017). The rate of cardiovascular adverse events in patients who received PLD-based combination therapy for NHL has not been studied adequately. The majority of people in this study were older than 60 years. The overall cardiovascular adverse event rate was 7.45%, indicating the safety of the (R)-CDOP regimen in the elderly population.

In the clinical use of chemotherapeutic agents, potentially lethal toxicity can lead to serious cardiovascular adverse events such as myocardial infarction, heart failure, cardiac arrest, and cardiogenic death. Therefore, we need to further investigate the severity of cardiovascular adverse events associated with (R)-CDOP (Caspani et al., 2021). Due to the differences in the incidence and severity of cardiotoxicity, a general tool is required to assess their severity, which includes the World Health Organization (WHO) and the Common Terminology Criteria for Adverse Events (CTCAE). Grade 3 and higher cardiovascular events often require immediate treatment (Atkinson et al., 2016). We classified cardiovascular events of grade 3 or higher, treatment discontinuation attributable to cardiovascular events, and death caused by cardiovascular events as serious cardiovascular adverse events. We found that the serious adverse cardiovascular event rate was 0.67%, including a heart failure rate of 0.55%, indicating that serious cardiovascular events are not likely to occur in clinical practice. Our study further found that the (R)-CDOP regimen had a 0.02% rate of treatment interruption attributable to left ventricular dysfunction or heart failure, and no deaths attributable to cardiovascular events occurred. This suggests that the (R)-CDOP regimen can significantly prolong patient survival by avoiding treatment interruption or death attributable to cardiotoxicity, thus providing greater benefit to patients.

The (R)-CDOP regimen is often used as an alternative to the (R)-CHOP regimen. However, the cardiac safety of the former regimen is unclear in the overall NHL treatment population. Further studies are still needed to determine whether the multidrug combinations increase cardiotoxicity. This study revealed that (R)-CDOP carried lower risks of total (OR = 0.161) and non-serious cardiovascular adverse events (OR = 0.171) than the (R)-CHOP group, implying a reduced risk of non-fatal cardiotoxicities, such as ECG abnormalities that do not require treatment, asymptomatic cardiac insufficiency, and minor abnormal cardiac laboratory results, for (R)-CDOP. Serious cardiovascular adverse events such as refractory heart failure or other difficult-to-control cardiac symptoms place a large burden on families and the entire healthcare system, which requires us to further explore the severity of cardiovascular adverse events. This study recorded lower risks of serious cardiovascular adverse events (OR = 0.252) and heart failure (OR = 0.294) for (R)-CDOP, providing evidence of its cardiovascular safety in the treatment of NHL. In addition, the meta-analysis of the efficacy suggested that R-CDOP might provide another option for patients with NHL.

To mitigate the occurrence of serious cardiovascular adverse events, cardioprotective agents have been used in combination with anthracyclines. Dexrazoxane is the only drug approved by the FDA for the prevention of cardiotoxicity (Yu et al., 2020). Some clinical trials are currently investigating cardioprotective agents for anthracycline-induced cardiotoxicities, such as beta-blockers and angiotensin-converting enzyme inhibitors. Caspani et al. noted that RAAS blockers, beta-blockers, and aldosterone antagonists provided a statistically significant benefit in the prevention of reduced left ventricular ejection fraction, but this positive effect remains to be confirmed (Caspani et al., 2021). The OVERCOME trial showed that patients with hematological tumors who took enalapril or carvedilol had a lower frequency of heart failure, LVEF <45%, or sudden cardiac death compared with the placebo group (Bosch et al., 2013). Therefore, the use of combination chemotherapy and prevention of chemotherapy-related cardiotoxicity will require a concerted effort by cardiologists and hematologists.

This meta-analysis provided evidence for the cardiac safety of (R)-CDOP combination for NHL, but several limitations must be noted. First, the definition of cardiovascular adverse events was not uniform in some of the studies, which may have affected the incidence of the reported adverse events. Second, single-arm studies include some heterogeneity, which may have affected the accuracy of the results. Finally, some of the included studies had small sample sizes, and the studies did not provide detailed classifications of cardiovascular adverse events.

## Conclusion

The (R)-CDOP regimen had a lower cardiovascular risk and was no less efficacious than (R)-CHOP in the treatment of NHL. This analysis suggested that (R)-CDOP therapy is useful in clinical practice and clinical reference. Due to the limitations regarding the number and quality of the included studies, these findings must be validated by additional high-quality studies.

## Data Availability

The original contributions presented in the study are included in the article/Supplementary Material; further inquiries can be directed to the corresponding authors.
