# Supplementary material for: Cardiovascular adverse events associated with cyclophosphamide, pegylated liposomal doxorubicin, vincristine, and prednisone with or without rituximab ((R)-CDOP) in non-Hodgkin’s lymphoma: A systematic review and meta-analysis
Source: Front Pharmacol. 2022 Dec 1;13:1060668. doi: 10.3389/fphar.2022.1060668 (PMC9752891; doi:10.3389/fphar.2022.1060668)
Supplement: Supplementary file 1 [file DataSheet1.docx]

# Search string

| **Pubmed** | |
| --- | --- |
| **Domain** | "Lymphoma"[Mesh] OR "Lymphoma, Non-Hodgkin"[Mesh] OR "Lymphoma, T-Cell"[Mesh] OR "Lymphoma, B-Cell"[Mesh] OR "Lymphoma, Mantle-Cell"[Mesh] OR "Lymphoma, Large B-Cell, Diffuse"[Mesh] OR "Lymphoma, Follicular"[Mesh] OR "Burkitt Lymphoma"[Mesh] OR “lymphoma" OR "Non-Hodgkin lymphoma" OR "T-Cell Lymphoma" OR "B-Cell Lymphoma" OR "Mantle-Cell lymphoma" OR "Diffuse Large B-Cell lymphoma" OR "DLBCL" OR "Follicular Lymphoma" OR "Burkitt Lymphoma" |
| **Determinant** | "R-CDOP"[tiab] OR "CDOP"[tiab] OR "CDOP protocol" [Supplementary Concept] OR "pegylated liposomal doxorubicin"[tiab] OR "PLD"[tiab] |
| **Specification** | Clinical Trial[ptyp] |

| **Embase** | |
| --- | --- |
| **Domain** | #1 'hematologic disease' OR 'leukemia' OR 'lymphoma' OR 'nonhodgkin lymphoma' OR 'chronic lymphatic leukemia' AND [embase]/lim |
| **Determinant** | #2 'cyclophosphamide plus doxorubicin plus prednisolone plus rituximab plus vincristine'/exp OR 'chop protocol'/exp OR 'R-CDOP'/exp OR 'CDOP'/exp OR 'CDOP protocol'/exp [Supplementary Concept] OR 'pegylated liposomal doxorubicin'/exp OR 'PLD'/exp AND [embase]/lim |
| **Specification** | controlled clinical trial'/de OR 'randomized controlled trial'/de |

**Cochrane Central Register of Controlled Trials**

| **Search term** | R-CDOP OR CDOP OR CDOP protocol OR pegylated liposomal doxorubicin OR PLD OR liposomal doxorubicin |
| --- | --- |

| **CNKI, Wanfang, and VIP databases** | |
| --- | --- |
| **Search term** | CDOP OR 脂质体 AND 淋巴瘤[题名或关键词] |

# Inclusion and exclusion criteria

| **Inclusion criteria** | **Exclusion criteria** |
| --- | --- |
| - People aged 18 years and over with non-Hodgkin's lymphoma - Treatment with (R)-CDOP combination regimen - If the article includes the control group, the control group is treated with the (R)-CHOP regimen and the experimental group is treated with the (R)-CDOP regimen - Outcomes containing cardiovascular adverse events | - Combining other chemotherapy drugs - **Liposomal doxorubicin is non-pegylated liposomal doxorubicin** - Full text is not available - Repeated publications - Reviews, animal studies, systematic reviews and case reports |

# Methodological index for non-randomized studies，MINORS

| **MINORS** | |  |
| --- | --- | --- |
| ①Clear research purposes | ⑦Less than 5% missed visits |  |
| ②Consistency in patient inclusion | ⑧Estimated sample size |  |
| ③Collection of prospective data | ⑨Appropriate selection of control group |  |
| ④The purpose appropriately reflected in the results | ⑩Concurrent control group |  |
| ⑤Objectivity in the evaluation of research indicators | ⑪Comparable baselines between groups |  |
| ⑥Adequate follow-up time | ⑫Appropriateness of statistical analysis |  |
| Items 9-12 are additional criteria to evaluate studies with a control group. Each item is scored from 0-2 out of 24. 0 means not reported, 1 means reported but insufficient information, 2 means reported and sufficient information provided. | | |

# 4. **Risk of bias analysis**

| **Reporting** | Low risk: events were reported clearly |
| --- | --- |
|  | High risk: events were not reported |
|  | Unclear: events were reported, but were not described in different treatment regimens separately |
| **Grading system** | Low risk: events were reported in a grading system |
|  | High risk: events were not reported in a grading system |
|  | Unclear: the study did not indicate whether events were reported in a grading system |
| **Completeness** | Low risk: events were reported in the whole population |
|  | High risk: events were reported only in a portion of the population |
|  | Unclear：the study did not indicate whether events were reported in the whole population |
| **Severity** | Low risk: the severity of events was reported |
|  | High risk: the severity of events was not reported |
|  | Unclear: the study did not indicate whether the severity of events was reported |
| **Specification** | Low risk: the specification of events was described in detail |
|  | High risk: the specification of events was not further detailed |
|  | Unclear: the specification of events was described unclearly |

# 5. The funnel plots of the publication bias

### Fig 2-funnel plot


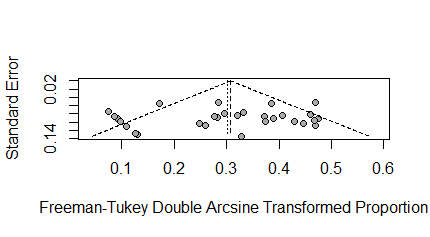


### Fig 3-funnel plot


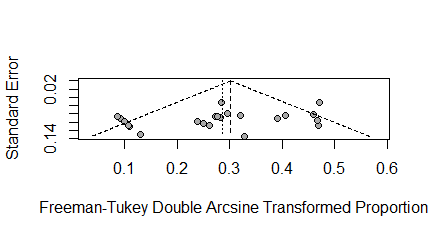


### Fig 4-funnel plot


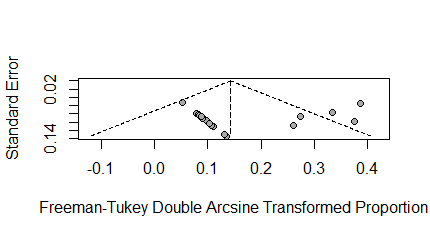


### Fig 5-funnel plot


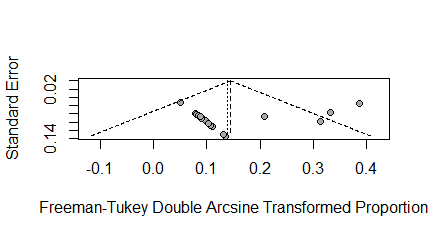


### Fig 6-funnel plot


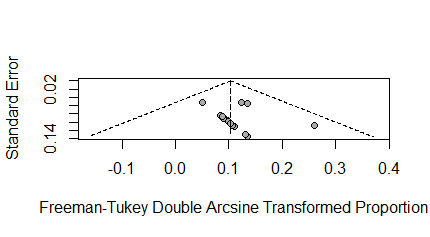


### Fig 7-funnel plot


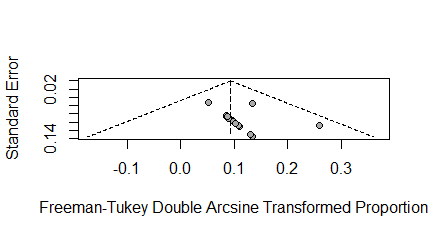


### Fig 8-funnel plot


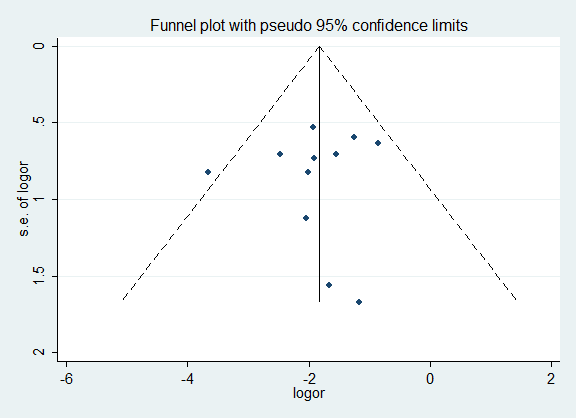


### Fig 9-funnel plot


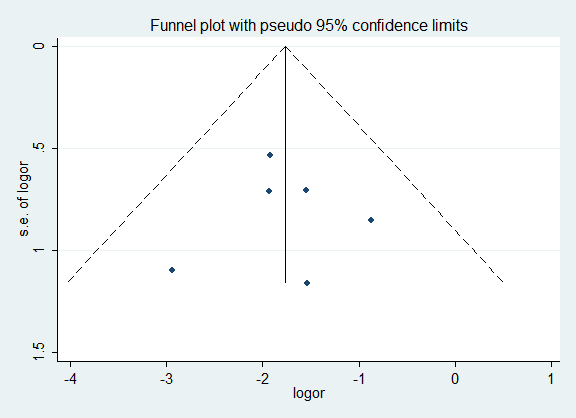


### Fig 10-funnel plot


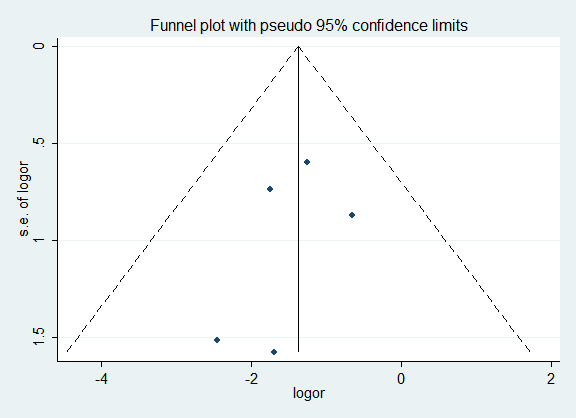


### Fig 11-funnel plot


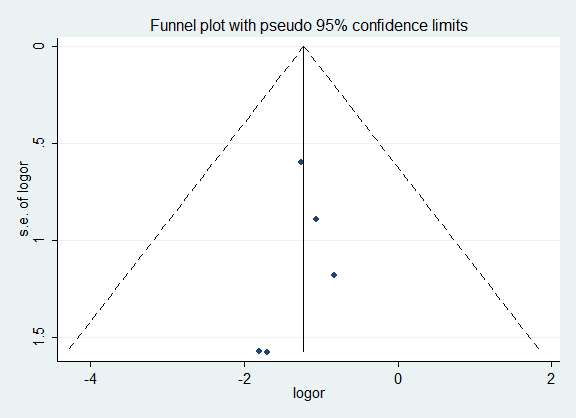


### Fig 12-funnel plot


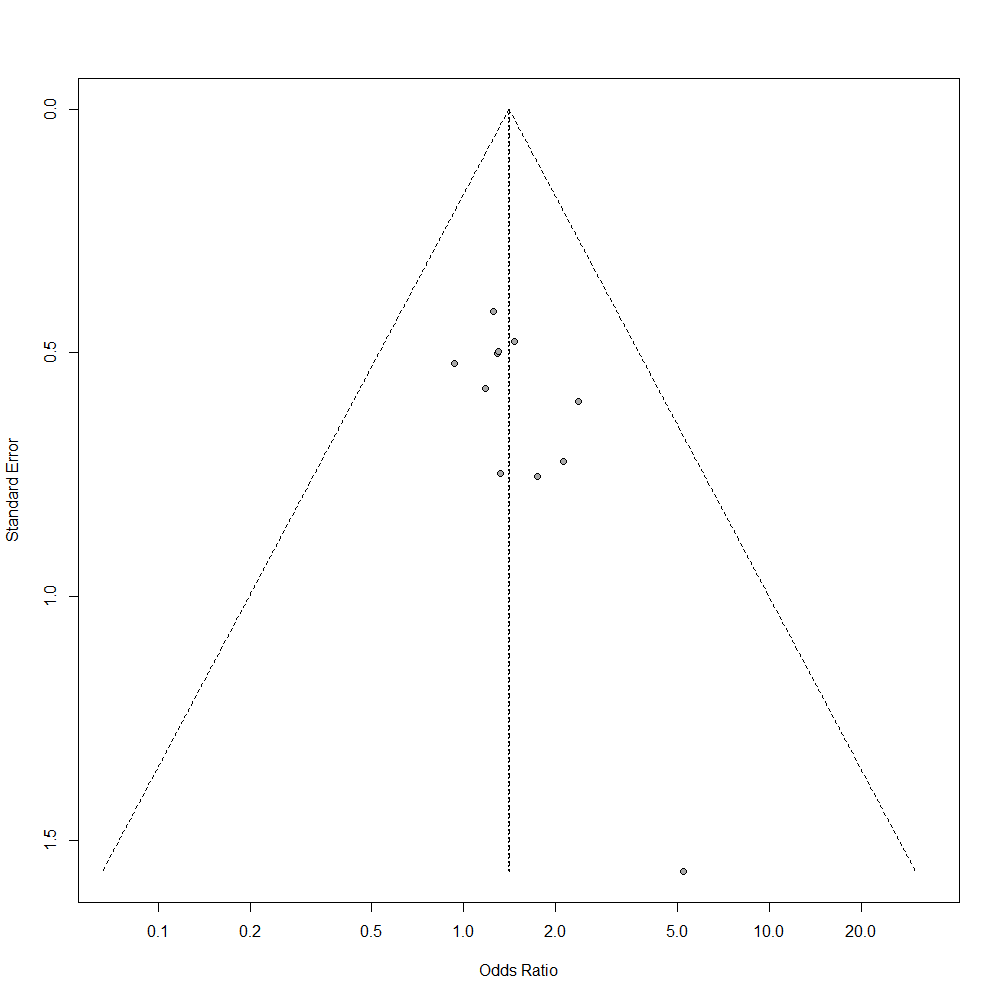


### Fig 13-funnel plot


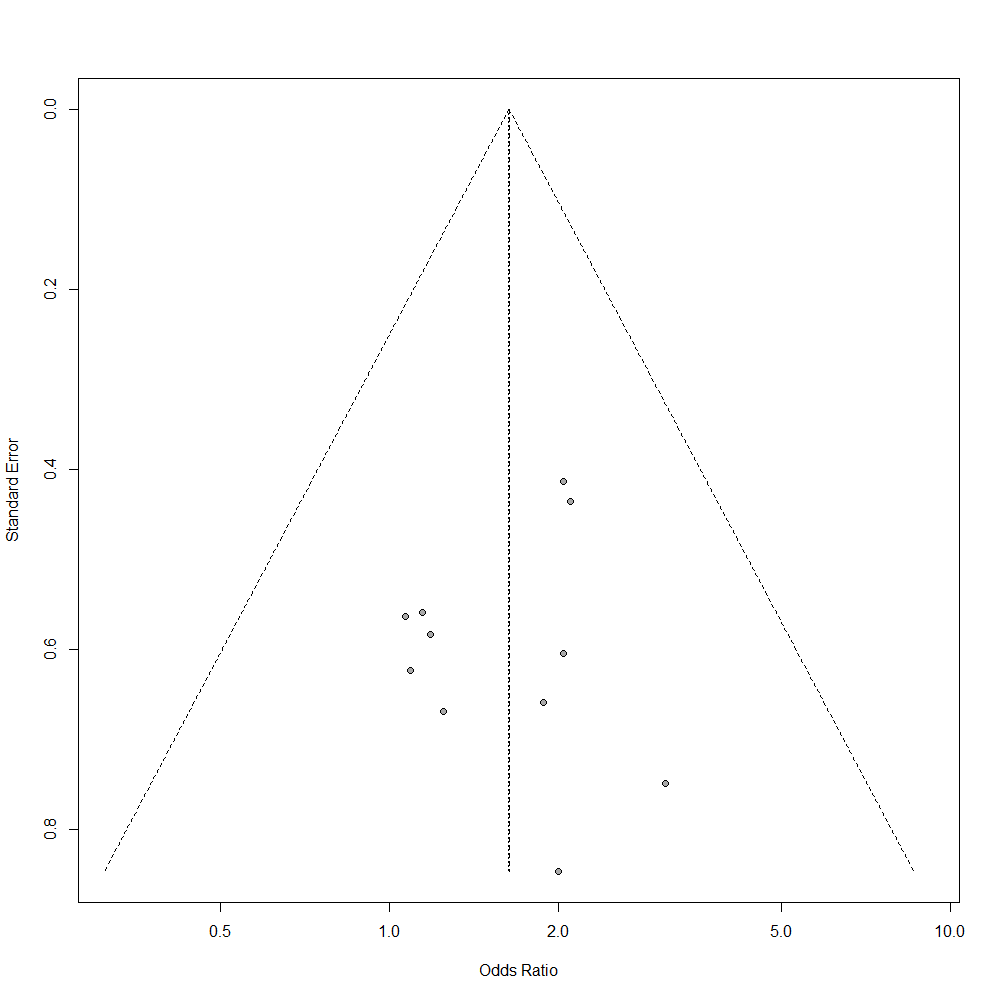


### Fig 14-funnel plot


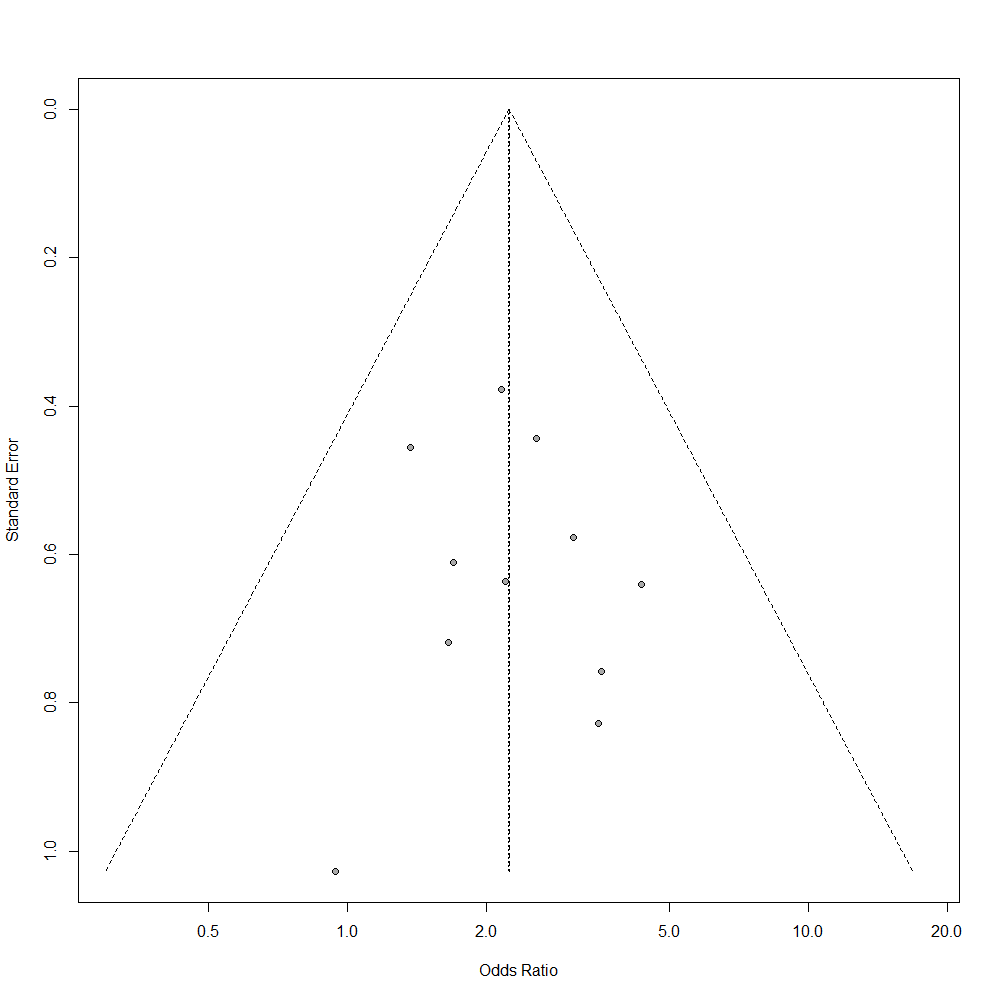


### Fig 15-funnel plot


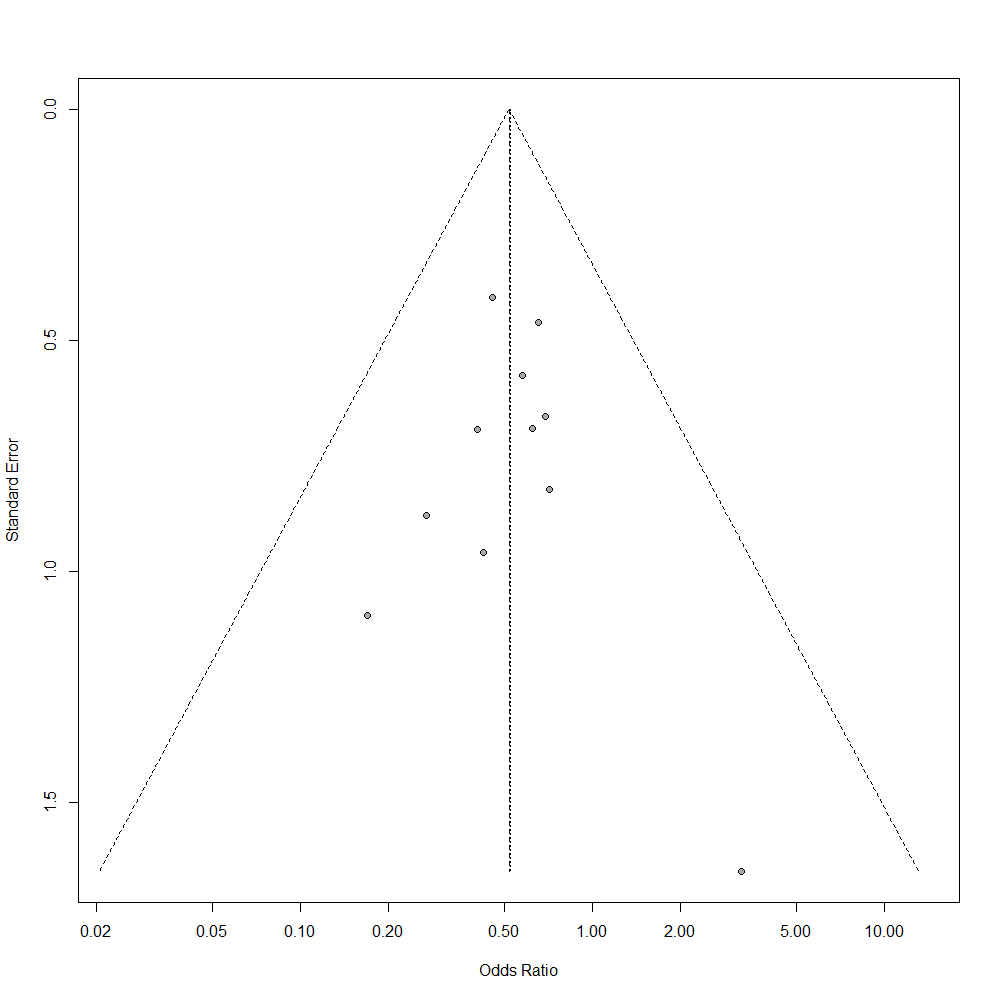


### Fig 16-funnel plot


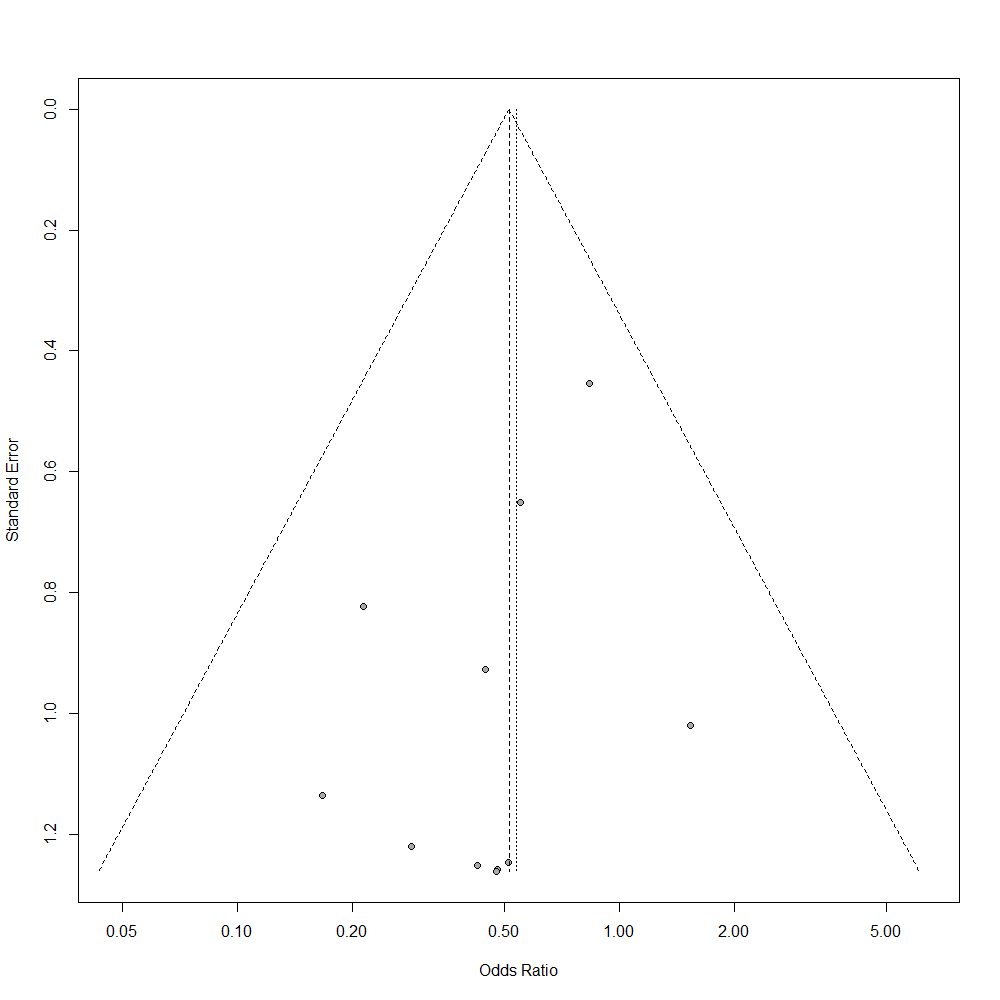


# 6.Assessment for all included studies

| **Author** | **1** | **2** | **3** | **4** | **5** | **6** | **7** | **8** | **Total** | **9** | **10** | **11** | **12** | **Total** |
| --- | --- | --- | --- | --- | --- | --- | --- | --- | --- | --- | --- | --- | --- | --- |
| **Liu, C.Y**  **2021** | **2** | **0** | **2** | **2** | **0** | **2** | **0** | **0** | **8** | **2** | **1** | **2** | **2** | **15** |
| **Zaja, F**  **2006** | **1** | **2** | **2** | **2** | **0** | **2** | **2** | **0** | **11** |  |  |  |  |  |
| **Martino, R**  **2002** | **2** | **2** | **1** | **2** | **0** | **2** | **2** | **0** | **11** |  |  |  |  |  |
| **Aviles, A**  **2002** | **2** | **2** | **2** | **2** | **0** | **2** | **2** | **0** | **12** |  |  |  |  |  |
| **Tsavaris, N**  **2002** | **2** | **2** | **2** | **1** | **0** | **2** | **2** | **0** | **11** |  |  |  |  |  |
| **Oki, Y**  **2015** | **2** | **0** | **2** | **2** | **0** | **2** | **2** | **0** | **10** |  |  |  |  |  |
| **Schmitt, C. J**  **2012** | **2** | **0** | **2** | **2** | **0** | **2** | **2** | **0** | **10** |  |  |  |  |  |
| **Zhou, D**  **2015** | **2** | **0** | **2** | **1** | **0** | **2** | **2** | **0** | **9** | **2** | **2** | **2** | **1** | **16** |
| **Visani, G**  **2005** | **2** | **0** | **2** | **2** | **0** | **2** | **2** | **0** | **10** |  |  |  |  |  |
| **Fan,Y**  **2011** | **2** | **0** | **2** | **2** | **0** | **2** | **2** | **0** | **10** |  |  |  |  |  |
| **Yang, F.L**  **2019** | **2** | **0** | **2** | **2** | **0** | **0** | **2** | **0** | **8** |  |  |  |  |  |
| **Shen, W.N**  **2016** | **2** | **0** | **2** | **1** | **0** | **2** | **2** | **0** | **9** |  |  |  |  |  |
| **Lin, X.Y**  **2020** | **2** | **0** | **2** | **2** | **0** | **2** | **2** | **0** | **10** |  |  |  |  |  |
| **Shao, Y**  **2020** | **2** | **1** | **2** | **2** | **0** | **2** | **2** | **0** | **11** | **2** | **2** | **2** | **2** | **19** |
| **Zheng, F.Y**  **2018** | **2** | **1** | **1** | **2** | **0** | **2** | **2** | **0** | **10** | **2** | **2** | **2** | **2** | **18** |
| **Hu, X.X**  **2018** | **2** | **1** | **2** | **2** | **0** | **2** | **2** | **0** | **11** | **2** | **2** | **1** | **2** | **18** |
| **Jia, C.M**  **2017** | **2** | **1** | **2** | **2** | **0** | **2** | **2** | **0** | **11** |  |  |  |  |  |
| **Shen, B**  **2020** | **1** | **1** | **2** | **1** | **0** | **1** | **2** | **0** | **8** | **2** | **2** | **1** | **2** | **15** |
| **Li, Z.H**  **2016** | **2** | **1** | **2** | **2** | **0** | **2** | **2** | **0** | **11** | **1** | **2** | **2** | **2** | **18** |
| **Huang, J.Q**  **2016** | **2** | **1** | **2** | **2** | **0** | **2** | **1** | **0** | **10** | **1** | **2** | **2** | **2** | **17** |
| **Huang, G**  **2021** | **1** | **1** | **2** | **1** | **0** | **2** | **2** | **0** | **9** | **1** | **2** | **2** | **2** | **16** |
| **Guo, B**  **2009** | **1** | **1** | **1** | **2** | **0** | **2** | **2** | **0** | **9** |  |  |  |  |  |
| **Wang, X.D**  **2021** | **2** | **1** | **2** | **2** | **0** | **2** | **2** | **0** | **11** |  |  |  |  |  |
| **Ye, J**  **2022** | **1** | **1** | **2** | **2** | **0** | **2** | **2** | **0** | **10** | **1** | **2** | **2** | **2** | **17** |
| **Chang, Y.Y**  **2021** | **2** | **0** | **2** | **2** | **0** | **0** | **2** | **0** | **8** | **2** | **2** | **2** | **2** | **16** |
| **Gui, L**  **2015** | **2** | **1** | **2** | **2** | **0** | **2** | **2** | **0** | **11** |  |  |  |  |  |
| **Li, J**  **2018** | **2** | **1** | **2** | **2** | **0** | **2** | **2** | **0** | **11** | **2** | **2** | **2** | **2** | **19** |
| **Wu, X.S**  **2020** | **1** | **1** | **2** | **2** | **0** | **2** | **2** | **0** | **10** | **1** | **2** | **2** | **2** | **17** |
| **Liu, S.R**  **2021** | **1** | **1** | **2** | **1** | **0** | **2** | **2** | **0** | **9** | **2** | **2** | **2** | **2** | **17** |
| **Song, X**  **2014** | **2** | **0** | **2** | **2** | **0** | **0** | **2** | **0** | **8** |  |  |  |  |  |

# **7.** **Risk of bias for all included studies**

| **Author** | **Reporting** | **Grading system** | **Completeness** | **Severity** | **Specification** |
| --- | --- | --- | --- | --- | --- |
| Liu, C.Y 2021 | Low | High | Low | Unclear | Low |
| Zaja, F  2006 | Low | Low | Low | Low | Low |
| Martino, R 2002 | Low | Low | Low | Low | High |
| Aviles, A 2002 | Low | Low | High | Low | Low |
| Tsavaris, N 2002 | Low | Low | Low | Low | Low |
| Oki, Y  2015 | Low | Low | Low | Low | Low |
| Schmitt, C. J 2012 | Low | Low | High | Unclear | Low |
| Zhou, D 2015 | Low | Low | High | Low | High |
| Visani, G 2005 | Low | Low | Low | Low | High |
| Fan,Y  2011 | Low | Low | Low | Low | High |
| Yang, F.L 2019 | Low | Low | Low | Unclear | Low |
| Li, Z.H  2016 | Low | High | High | Unclear | Low |
| Shen, W.N 2016 | Low | Low | High | Low | Low |
| Lin, X.Y 2020 | Low | Low | Low | Low | Low |
| Shao, Y 2020 | Low | High | Low | Unclear | Low |
| Zheng, F.Y 2018 | Low | High | Low | Unclear | Low |
| Hu, X.X 2018 | Low | High | Low | Unclear | High |
| Jia, C.M 2017 | Low | Low | Low | Low | Low |
| Shen, B  2020 | Low | High | Low | Unclear | High |
| Huang, J.Q 2016 | Low | Low | Low | Unclear | High |
| Huang, G  2021 | Low | High | Low | Unclear | High |
| Guo, B  2009 | Low | Low | Low | Unclear | Low |
| Wang, X.D 2021 | Low | Low | Low | Low | Low |
| Ye, J  2022 | Low | High | Low | Unclear | High |
| Chang, Y.Y 2021 | Low | Low | Low | Unclear | Low |
| Gui, L  2015 | Low | Low | Low | Low | Low |
| Li, J  2018 | Low | Low | Low | Low | Low |
| Wu, X.S 2020 | Low | Low | High | Low | Low |
| Liu, S.R 2021 | Low | High | Low | Unclear | High |
| Song, X 2014 | Low | High | High | Low | Low |

# 8. **Risk of bias for all included studies-Overview**
